# Modeling the social determinants of resilience in health professions students: impact on psychological adjustment

**DOI:** 10.1007/s10459-023-10222-1

**Published:** 2023-05-16

**Authors:** Ross Perry, Andres Sciolla, Margaret Rea, Cara Sandholdt, Karl Jandrey, Elizabeth Rice, Allison Yu, Erin Griffin, Michael Wilkes

**Affiliations:** 1 School of Medicine, University of California, Davis, Sacramento, CA USA; 2 Department of Psychiatry and Behavioral Sciences, University of California, Davis, Sacramento, CA USA; 3 Betty Irene Moore School of Nursing, University of California Davis, Sacramento, CA USA; 4 School of Veterinary Medicine, University of California, Davis , Davis, CA USA; 5 Washington State University, Elson S. Floyd College of Medicine, Spokane, WA USA

**Keywords:** Health professions education, Resilience, Veterinary medicine students, Nursing students, Medical students, Learning environment, Mental health, Disadvantaged students

## Abstract

Stressors inherent to training and stemming from the learning environment are associated with high rates of burnout, depression, and mental health problems in health professions students (HPS). There is evidence that disadvantaged or stigmatized groups are particularly affected. These problems not only impact students after graduation but may also have detrimental effects on patient outcomes. Resilience, conceptualized as the process of adapting well in the face of adversity, has inspired an increasing number of interventions aimed at addressing those problems in HPS. These interventions have mostly targeted individual students and their psychological traits while ignoring social and structural factors that may enhance or undermine individual resilience. To address this gap in the literature, the authors reviewed the evidence for psychosocial determinants of resilience and proposed a model inspired by the social determinants of health literature and the “upstream–downstream” metaphor. In this theoretical paper, the authors propose that upstream determinants such adverse childhood experiences and socioeconomic and sociodemographic markers of disadvantage have a direct effect on psychological adjustment and an indirect effect mediated by resilience. Additionally, the authors propose that the institutional downstream drivers of learning environment, social support, and sense of belonging moderate the direct and indirect effects of the upstream determinants on psychological adjustment. Future research should test these hypotheses and gather evidence that may guide the development of interventions. The authors present their model as part of a comprehensive response to recent calls to action to address diversity, equity and inclusion in health professions education.

## Introduction

Health professions education takes place in stressful environments with high academic demands, substantial workloads, and increased psychological pressure (Collins & Foote, 2005; Fares et al., 2016; Labrague et al., 2017). Health professions students (HPS) experience higher rates of burnout and depression than their age-matched peers, despite entering professional education with relatively lower psychological distress (Dyrbye et al., 2010a; Killinger et al., 2017; Rotenstein et al., 2016). Poor mental health in HPS has been associated with low standardized test scores, professionalism lapses, and dropout (Jackson et al., 2016; Strauss et al., 2019). Worse yet, burnout has deleterious effects on healthcare quality and safety (Dyrbye et al., 2010a; Panagioti et al., 2018; Rudman & Gustavsson, 2012). Among the “calls to action” to address the harmful effects of stress on HPS, interventions targeting psychological resilience have gained considerable traction.

Resilience is variously defined as the “dynamic capability which can allow people to thrive on challenges given appropriate social and personal contexts (Howe et al., 2012)” and an active adaptation to stress resulting from biopsychosocial and ecological interactions (Kalisch et al., 2017). Regarding psychological adjustment, studies in HPS trainees and professionals have shown that resilience is positively associated with psychological well-being and negatively associated with burnout, anxiety, and depression (Chow et al., 2018; Kunzler et al., 2020; Mealer et al., 2012; Rees et al., 2015; Yu & Chae, 2020). Many modifiable determinants of resilience at the *individual* level have been validated, including meaning in life, sense of coherence, hardiness, self‐esteem, active coping, self‐efficacy, cognitive flexibility, and religiosity (Kunzler et al., 2020). While determinants of resilience are indeed important, the emphasis on resilience as an individual trait has placed the onus of “resilience failures” on individual HPS (Schoon & Bynner, 2003). This overshadows a *systemic* view in which “resilience depends just as much on the culturally relevant resources available to stressed individuals in their social, built, and natural environments as it does on individual thoughts, feelings, and behaviors” (Ungar & Liebenberg, 2011).

**Fig. 1:**
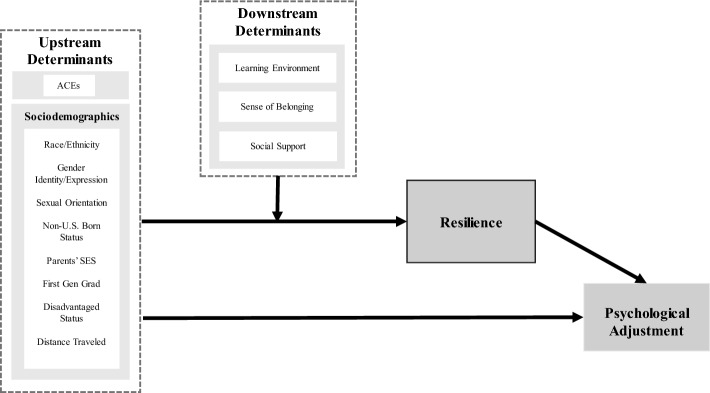
The social determinants of resilience in health professions students model

Systemic and institutional determinants may compound the impact of adversities that HPS may have faced before matriculation on resilience and psychological adjustment. Systemic and structural factors have been well-researched in the context of the social determinants of health (SDOH). According to SDOH models, a significant percentage of adult health outcomes are attributable to economic policies and systems, developmental agendas, social norms, social policies and political systems (Commission on Social Determinants of Health, 2008). In SDOH models, a well-known metaphor differentiates upstream (“the cause of causes”) from downstream determinants (proximal causes) of health disparities (Braveman & Gottlieb, 2014). Generally, upstream determinants include socioeconomic resources, education, and racial discrimination while downstream determinants include health-related behaviors, disease, and injury (Braveman & Gottlieb, 2014). Based on a careful literature review and discussions by a multidisciplinary group of educators and social scientists, we proposed a model applying “social determinants of resilience” and created a visual representation (Fig. 1).

## Method

Drawing from SDOH models, we conducted a targeted literature review of peer-reviewed articles in English discussing psychological constructs related to resilience that could be measured with brief and validated measures that had been used in veterinary, nursing and medical students, which are the three health professional schools at our university. Of note, our research project is one among several initiatives that seek to address diversity, equity and inclusion at our institution. The short-term aim of our project is to test our model with a confidential, online survey containing those measured administered prospectively to HPS over the course of their schooling. The long-term goal is to build an evidence base that may help develop and test equity-focused and resilience-informed interventions to enhance well-being and academic progress in all students.

We initially limited our search to the last 30 years; however, we allowed exceptions when identified articles referenced important work published before our original cutoff. The results of the review were extensively discussed by a multidisciplinary team of HPS and educators in an iterative process taking place over a period of several months.. We hypothesized that these psychological constructs and their measures would comprise the downstream determinants of resilience and psychological adjustment in our model. In a few cases where the evidence in HPS was scant, we searched the literature in college students for evidence supporting our model’s hypotheses. Since we found evidence that these constructs have independent effects on both resilience and psychological adjustment, we further hypothesized that resilience would partly mediate these effects.

We also posited that upstream determinants should include early life stress and sociodemographic characteristics associated with social disadvantage.

## Results

### Upstream determinants of resilience

We begin with upstream drivers, a logical starting point within this model, as these determinants entail the “causes of causes” within classic models of SDOH (Braveman & Gottlieb, 2014). In SDOH models, restricted outcomes over one’s lifetime and across generations may lead to psychological maladaptation and an accumulation of risk as one risk factor reinforces others (Schoon & Bynner, 2003), Huang et al. (2015). Viewing resilience from this perspective, social determinants impact HPS long before they receive HPS education, leading to negative outcomes. For example, in a study of over 2000 pre-nursing university students, most students lost prior to nursing program application were first generation or minority students, an example of the so-called “leaky pipeline” (Bennett et al., 2020). Just as SDOH research has shown that addressing social determinants of health can lead to reductions in health disparities (Braveman & Gottlieb, 2014), research on our model may lead to innovative interventions to enhance resilience in HPS.

### Adverse childhood experiences

Adverse childhood experiences (ACEs) refer to childhood maltreatment, household and community dysfunction and other adversities experienced before the age of eighteen. The effects of self-reported ACEs on adult health have been well-documented, showing a graded relationship between the number of ACEs and health-harming behaviors and physical and mental health problems (Felitti et al., 1998).

In educational settings, ACEs exposure has been associated with declining educational achievement, worsening mental health, and increased dropout rates in college students (Duncan, 2000; Karatekin, 2018). Among nursing, medical and veterinary medical students, studies show over half reporting at least one ACE (Kameg et al., 2020; Sciolla et al., 2019; Strand et al., 2017). In studies conducted with nursing and medical students, higher numbers of ACEs were associated with increased risk for depression and burnout (McKee-Lopez et al., 2019).

Starting in the second half of the twentieth century, the relationship between childhood maltreatment and resilience (and subsequently ACEs and resilience) has received increased attention by clinicians, researchers and policy makers. Scholars argue that women’s rights in the Western world, which led to a shift in society’s view of children’s agency, provided a powerful impetus for that interest (Haring et al., 2019). For example, higher levels of resilience have been shown to mitigate the negative impact of ACEs in children (Bethell et al., 2014). For HPS, evidence suggests ACEs-affected students are particularly at risk for training-related re-traumatization, highlighting a need for ACEs-informed training environments (Butler et al., 2017). Thankfully, the work has already begun, with thematic analysis of ACEs-affected medical students revealing several protective factors for resilience: non-parental support, role models, volunteering, and use of mental health services (Blickenstaff et al., 2021).

### Socioeconomic disadvantage

Socioeconomic disadvantage (SED) in the form of inequalities in income distribution is widely accepted as one of the most influential variables among the social determinants of health (Angell, 1993). With regards to mental health alone, SED accounts for a two-fold increase in mental health conditions between the lowest and highest socioeconomic brackets (Holzer et al., 1986). The literature reviewed revealed scant data for HPS, but one study of Chinese medical students found that the mother’s socioeconomic status (SES) (but not the father’s) was associated with the student's self-reported psychological distress (Fan et al., 2007).

While research suggests low SES is associated with resilience deficits among national adult cohorts (Cosco et al., 2018), we are not aware of studies that have explored the connection between these variables among HPS. One study of ninth-grade students in a national probability sample found that a fixed mindset (a proxy for lower resilience in an expanded version of our model; see Limitations) was more prevalent in low SES students (Destin et al., 2019). In developing the current model, we aim to advance an understanding of the relationship between socioeconomic disadvantage, resilience, mental health and academic outcomes, and inspire the development of resilience interventions that consider the needs of students from disadvantaged backgrounds.

### Sociodemographic disadvantage

*Race & Ethnicity:* In recent years, health professions educators have received calls to action to recruit and support a more diverse workforce (Elmore, 2003). Despite these calls, significant grading disparities have been found to favor white medical students over students who are underrepresented in medicine (URM) and non-URM minorities, even when adjusting for age, gender, maternal education, and standardized test scores (Low et al., 2019). Similarly, a review of studies involving nursing students found evidence suggesting that discrimination, bias in grading practices, and isolation may explain the higher attrition rates of minority nursing students as compared to white students (Graham et al., 2016). There is a paucity of research exploring the experiences of non-U.S. born citizens in pre-clinical training, but similar themes of isolation, insensitivity, and other challenges have been reported in international medical residents (Chen et al., 2011).

Research examining psychological adjustment in minority medical students suggests a complex relationship. Regarding differences in quality of life, depression, and burnout between URM and non-URM students, several large multicenter surveys show contradictory findings. Some African American medical student cohorts have reported greater burnout compared to non-URM students, while others have reported less burnout (Dyrbye et al., 2006, 2007). As our model will suggest, there may be another side to the story, as URM students appear to experience fewer mental health difficulties within supportive learning environments (Dyrbye et al., 2007). It is possible that URM students have their own “brand” of resilience, one best facilitated within learning environments aimed at supporting a more diverse student body.

*First Generation Status.* Thus far, most research among first generation students (i.e., those who are the first in their families to attend college) has been conducted in undergraduate populations, although there are a few examples among HPS. In one study of medical students, first-generation students had significantly lower scores on self-care, perceptions of medical school support for family and personal responsibilities, and higher scores on sleep problems when compared to continuing generation peers (Mason et al., 2018). Among college students, comparative data suggests that first-generation students tend to report lower ratings of belonging, greater levels of depression and stress, and lower use of services compared to continued-generation peers (Stebleton et al., 2014).

*Gender* There are remarkably few studies exploring gender difference in HPS in contrast to epidemiological data documenting gender differences across the lifespan in resilience, types of stressors and traumatic experiences, coping styles, and prevalence of stress-related disorders. For example, women and girls generally exhibit greater vulnerability (Hirani et al., 2016). However, several studies have found lower levels of resilience in female as compared to male HPS, although some studies show the opposite pattern (Sundar & Archana, 2020; Jordan et al., 2020; Bahadir-Yilmaz and Oz, 2015). Research focusing on gender differences in resilience has been criticized for neglecting social domains associated with resilience in women (Hirani et al., 2016). In line with this critique, a recent study of frontline health care workers responding to the COVID-19 pandemic measured contextual factors (i.e., background stressors such as pre-pandemic burnout and index stressors such as caring for children/dependents during the pandemic) in addition to stress-related psychological distress. After controlling for background and index stressors, the gender differences in psychological distress were no longer significant (Lowe et al., 2021). Similarly, empirical tests of our model should clarify the contribution of upstream and downstream determinants (i.e., background and index stressors) by controlling for them statistically, should they identify gender differences in resilience or psychological adjustment.

*Non-heterosexual sexual orientation and nonbinary gender identity.* This constitutes another example where the intersection of unsupportive learning environments and lifelong discrimination may result in adverse outcomes for HPS. In one of the largest longitudinal studies of its kind, medical students who identified themselves other than heterosexual reported higher levels of depression and anxiety than their heterosexual peers, a disparity that continued into residency training (Wang et al., 2020). Importantly, the association between non-heterosexual identity and psychological distress was partly mediated by experiences of harassment and isolation for medical students (Przedworski et al., 2015; Samuels et al., 2021) and a decreased sense of belonging for medical residents (Wang et al., 2020). Transgender medical students and physicians report censorship of speech or mannerisms and experiences of derogatory comments about transgendered individuals (Dimant et al., 2019). Thus far, research exploring resilience expression among LGBT + students across different learning environments appears practically non-existent. In developing the current model, we aim to promote further research in this important area of HPS well-being.

## Downstream determinants of resilience

Following SDOH models, downstream determinants of resilience in our model entail individual-level psychological and behavioral traits such as “adaptive coping” or “mindfulness” (Rees et al., 2015). In our model, we propose an additional set of downstream determinants that include systemic factors not experienced by HPS until after matriculation, such as the learning environment. Due to space limitations, we set aside a discussion of individual-level determinants to focus on structural and interpersonal determinants embedded in educational programs, which we propose determine the differential expression of resilience across different groups of students. In doing so, we aim to stimulate resilience research that is simultaneously broader and more targeted, encouraging interventions to foster resilience at the system level and not exclusively at the individual level, thus ensuring that those efforts are directed toward a diversity of student backgrounds.

In what follows, we focus on three important aspects of health professions education: learning environment, social support, and sense of belonging. Together, these variables make up the “culture” of a health professions institution and therefore determine resilience expression in our model in the same broad way that culture may determine health outcomes in SDOH models (Hafferty, 1998, Wilkes et al., 2013).

### Learning environment

Learning environment or climate is a broad term that encompasses physical and virtual classroom spaces, but also social and cultural contexts in which students learn (Genn, 2001). For HPS, the learning environment includes formal, informal, didactic, and clinical learning spaces, as well as sociocultural dimensions created by student, faculty and systemic factors. Learning environment can have both negative and positive effects on student achievement, engagement, motivation, and well-being (Soemantri et al., 2010).

Studies have shown that students from disadvantaged or stigmatized backgrounds may be particularly affected by their learning environment (Dyrbye et al., 2006, 2007; Samuels et al., 2021). In one study, multivariate analyses found a clear dose–response relationship between satisfaction with the learning environment and burnout, after controlling for clinical rotation characteristics and workload (Dyrbye et al., 2010b). Additionally, multisystem-based studies of health profession schools suggest that students, particularly URM students, continue to experience both overt and subtle manifestations (i.e., microaggressions) of negative learning environments in clinical programs (Ackerman-Barger et al., 2020). For example, URM medical students experience significantly more stereotype threat than age-matched peers, which has been shown to correlate with fewer honors earned in third-year clerkships (Bullock et al., 2019).

In U.S. medical students, higher resilience is significantly correlated with a more positive perception of the learning environment (Dyrbye et al., 2010b), while research in nursing students indicates that the impact of individual resilience on psychological and academic distress may be negatively amplified in unsupportive learning environments (Mcdermott et al., 2020). Together, these results suggest that learning environments that are supportive of the diverse needs of students may level the playing field and increase the chances that everyone can succeed, not just students with resilient personality traits (Mcdermott et al., 2020). URM nursing students report that increasing faculty diversity has been key to improving learning environment inclusivity (Metzger et al., 2020). This suggests that structural interventions may be essential for facilitating resilience among a diverse health professions student body.

### Social support

Social support is a multidimensional construct highlighting the assisting nature of personal relationships, formally defined as the supply of tangible or intangible resources individuals gain from their network members (Song et al., 2011). There is evidence that social support has both direct and indirect effects on student well-being through moderation effects on stressors (e.g., academic load) and strain (e.g., student burnout). For example, a meta-analysis of the relationship between social support and student burnout reveals a strong, negative correlation (Kim et al., 2018). Likewise, studies in HPS have identified social support’s role in protecting psychological well-being and reducing perceived stress, depression, anxiety, and suicidal ideations (He et al., 2018; Mao et al., 2019; Park et al., 2015; Reeve et al., 2013). Research suggests a positive correlation between social support and resilience, particularly among URM students (Ozsaban et al., 2019; Acheampong et al., 2019).

### Sense of belonging

Sense of belonging refers to personal experience or involvement in a group or environment in which individuals perceive themselves as an integral part of that group or environment. A sense of belonging represents a fundamental human motivation, which is considered paramount to the human condition, often taking precedence over other needs, such as esteem and self-actualization (Baumeister and Leary, 1995). Acceptance and inclusion to a group, or lack there-of, may not be granted automatically, and may be communicated through subtle cues (Howansky et al., 2021). This aspect of belongingness may partially underpin the power of microaggressions to affect HPS, which –similarly to implicit bias—are considered subjective and unconscious, and therefore offenders may not view them as damaging (Turner et al., 2021).

Sense of belonging has been shown to be empirically important in student populations. In college students, greater sense of belonging is associated with less interpersonal conflict, loneliness, anxiety, and depression (Hagerty et al., 1996). Qualitative and quantitative studies in HPS have shown a significant impact of sense of belonging on choice of medical specialty, school retention, motivation to learn, and perceived stress (Gerull et al., 2021; Grobecker, 2016; Honda et al., 2016; Middleton et al., 2021; Reilly and Fitzpatrick, 2009). Of particular concern, URM medical students report a lower sense of belonging than age matched peers (Isik et al., 2021). We notice here a parallel with research testing minority stress mode, initially developed to understand mental health disparities affecting sexual minorities (Meyer, 2003). For example, in a recent study in higher education, nonbinary students reported high levels of minority stress, which was related to negative perceptions of belonging and learning environment (Budge et al., 2020). Promoting a better sense of belonging may be a key to facilitating improved resilience among diverse HPS, as greater sense of belonging in other groups has been consistently associated with psychological well-being and positive psychosocial outcomes (Allen et al., 2021).

## A model of the social determinants of resilience

The evidence reviewed suggests that measuring the effect of structural determinants of resilience may be critical in promoting positive psychological adjustment within diverse HPS, and avoiding deleterious outcomes such as depression, anxiety, burnout, and academic failures. Previous models of resilience have focused on individual determinants but have not accounted for the powerful influence of social and structural determinants within the life course of HPS. To bridge various levels of explanation, Fig. 1 depicts a model of resilience in HPS that takes advantage of conceptualizations underlying SDOH models. For heuristic purposes, the model places determinants along an upstream–downstream axis that converges on resilience to exert effects on psychological adjustment.

Upstream determinants are quantifiable markers of inequality in stress exposure and resources that may buffer against them. Among several to choose from, we have included ACEs, sociodemographic variables (e.g., race/ethnicity, gender, sexual orientation and gender identity/expression), and SED variables, which may influence resilience development before, during, and after matriculation to health professions programs. When testing our model, we intend to use multiple SED variables, depending on what is available to each health profession program. For example, medical schools can use the disadvantaged applicant status, a voluntary self-identification within in the American Medical College Application Service. The issues surrounding this particular variable (Lowrance and Birnbaum, 2019) suggest that no single SED variable (e.g., first-generation status or family’s SES) may fully explain the SED construct, hence the need to combine multiple variables whose face validity made the suitable to measure SED.

The model’s main variables are linked by two pathways in Fig. 1: (1) A direct influence of upstream variables upon mental health and psychological adjustment, and (2) an indirect pathway mediated by resilience. We propose that resilience, and therefore positive psychological adjustment within health programs, may vary widely based on the systemic, cultural, and economic pressures that play upon individuals differently according to each student’s race/ethnicity, SOGIE, and other sociodemographic characteristics.

The relationship between these upstream variables, resilience, and psychological adjustment may in turn be affected by downstream determinants built into institutional cultures (i.e. learning environment, social support, and sense of belonging). As reviewed, research indicates that each of these downstream determinants are correlated with both psychological adjustment and resilience. Furthermore, it has been shown that URM students are more likely to experience more unsupportive learning environments, less social support, and less sense of belonging than their peers (Dyrbye et al., 2007, 2010b; Isik et al., 2021; Samuels et al., 2021). Therefore, we predict two effects of downstream drivers: (1) a moderating effect on the pathway between upstream drivers and psychological adjustment, and (2) a moderating effect on the pathway from upstream drivers to resilience. In other words, the learning environment, social support, and sense of belonging should influence the psychological success of students differently, depending on their life courses and backgrounds. Moreover, individual resilience resulting from each student’s social determinants will be expressed most robustly within programs that best support them (i.e., programs with supportive learning and social environments and a high levels of inclusivity).

## Discussion

In response to rising rates of burnout and other mental health problems in HPS, educators and school administrators have focused on single or, less often, multiple individual personality traits. We argue that this approach represents a missed opportunity to address systemic or structural determinants of resilience. Here we address this gap with a focused review of the literature of those determinants and a resulting theory-driven, testable model. The model’s central premise is that by addressing systemic determinants of resilience, such as the learning environment, social support, and sense of belonging, psychological adjustment will improve for all students. The implications of this for health sciences educators is to approach learners with a richer, more targeted approach to promoting academic success. Critically, our model suggests that system-level interventions should be explored in addition to individual-level interventions, particularly for students from backgrounds historically marginalized and underrepresented in health professions.

By the very fact of matriculating in health professions schools, HPS from disadvantaged backgrounds have demonstrated the “capability to thrive on challenges given appropriate social and personal contexts”, which is the very definition of resilience (Howe et al., 2012). Our model was prompted by anecdotal observations and in-depth conversations with students who often struggle to navigate the stressors of health professions schools, despite a demonstrated ability to overcome adversity.

Perhaps, as health professions educators, we have erred in defining resilience quantitatively (i.e., some have less, some have more). Instead, we might ask what *type* of resilience a student may have, and what resources can best support the expression of these different “brands” of resilience. If the aim of health professions education is to build a resilient *and* diverse workforce, then it is our responsibility to understand how resilience is differentially expressed in students from diverse backgrounds, characterize the learning environments and supports that best foster resilience in all students, and rigorously translate that knowledge into institutional resources, policies, and professional and institutional cultures that promote a multilevel view of resilience.

By utilizing the present model, researchers may better aim investigations and interventions at determinants that are most relevant to resilience in diverse HPS, particularly disadvantaged students who face greater threats to optimal psychological adjustment and academic success. Beyond graduation, enhanced resilience in HPS may have a catalytic effect on future practice by lowering the risk of mental health problems (e.g., burnout) while maximizing outcomes known to improve patient experiences (e.g., reduced clinician turnover).

### Limitations and future directions

An important limitation of this proposal is that it is not the result of a systematic review of the literature but the product of extensive interdisciplinary dialogue, collective reflection, and a focused review of the literature. In addition, we focused our research on nursing, veterinary and medical students assuming that our model would be largely applicable to other HPS including physical and occupational therapy. Despite this caveat, the scope of our review was considerable: approximately two thirds of the articles reviewed were not included in the final manuscript. We feel confident that we are unlikely to have missed large empirical studies on the topic. While more comprehensive than most, an additional limitation of the current model is the exclusion of individual-level determinants (e.g., adaptive coping style and trait mindfulness) and extra-institutional structural determinants of resilience in HPS (e.g., precarious living environment, food insecurity, and accumulated debt) due to space limitations. Also absent from our model are microaggressions as well as disability status and other equity-denied groups and their intersections, which we view as structural determinants of resilience. While there is evidence linking racial discrimination to negative mental health outcomes and well-being in medical students (Perry et al., 2016), to our knowledge the interaction of those variables with resilience has only been shown in African American college students (Brown and Tylka, 2011) and registered nurses (Byers et al., 2021).

Since our reviewed targeted the empirical literature on resilience and its determinants, we did not discuss in any length critical conceptualizations of resilience that have been proposed by others (Luthar et al., 2000; Kalisch et al., 2017; Traynor, 2018; Ungar et al., 2013). Ours, however, seems to be the first of its kind as applied to HPS.

Another important limitation of this review and model is the focus on psychosocial to the exclusion of biological determinants. Future research may be able to integrate social and psychological aspects of resilience to the staggering body on data on neurobiological (Bush & Roubinov, 2021) and genetic (Niitsu et al., 2019) determinants of resilience. As an example of this type of research, we note a longitudinal study of first-year medical residents that found that saliva-measured telomere shortening (a biomarker of stress exposure) after a year was six times greater than expected and that shortening was associated with work hours (Ridout et al., 2019). Of significance to our model of upstream determinants, reported ACEs were significantly associated with baseline telomere length, after controlling for age and sex. If replicated, a corollary to this type of study is that the effectiveness of future structural or systemic as well as individual-level interventions may be tracked by non-invasive resilience biomarkers, in addition to self-reported and interviewer-based measures of resilience and psychological adjustment.

Another line of inquiry for future research is the inclusion of academic success as an additional outcome in our model, in addition to psychological adjustment. The literature of the relationship between resilience and academic performance in HPS is equivocal (Chisholm-Burns et al., 2021), although studies in college students in the U.S. and elsewhere consistently find an association between resilience and academic success (Hartley, 2011; Bittmann, 2021; Allan et al., 2014).

Our model is currently being tested at the University of California, Davis School of Medicine, School of Veterinary Medicine, and Betty Irene Moore School of Nursing. Starting in the fall of 2019, our research team has been conducting a longitudinal study to assess changes in perceptions of learning environment and social support over time and correlating these changes with measures of resilience, mental health, and academic achievement. Through this interdisciplinary approach, we hope to verify the accuracy of our theory-based model and advance the general understanding of the social determinants of resilience in HPS.

## Conclusion

Considering gaps in the literature of resilience in HPS and a critical appraisal of the construct, the authors reviewed the evidence for psychosocial determinants of resilience and propose a model inspired by the SDOH literature and its well-known “upstream–downstream” metaphor. Although the model is consistent with similar conceptualizations of resilience by others, to our knowledge this is the first application to HPS. The authors propose that upstream determinants have a direct effect on psychological adjustment and an indirect effect mediated by resilience. The authors also propose that the each of the downstream drivers learning environment, social support, and sense of belonging moderate the direct and indirect effects of the upstream determinants on psychological adjustment. Unquestionably, the value of our model awaits empirical testing by our group (and hopefully others). Meanwhile, this cohesive set of hypotheses grounded in available research represents a cogent response to calls to action to address both stress-related psychological problems in HPS as well as long-standing systemic barriers to achieve equity, diversity and equity in health professions education. We hope that evidence gathered that may inform the development of interventions targeting systemic, institutional and individual determinants that undermine resilience and academic success at the undergraduate and graduate levels of health professions education.
